# Analysis of Epstein Barr Virus Encoded RNA Expression in Nasopharyngeal Carcinoma in North-Eastern India: A Chromogenic in Situ Hybridization Based Study

**Published:** 2016-07

**Authors:** Anjan Saikia, Vandana Raphael, N-Brian Shunyu, Yookarin Khonglah, Jaya Mishra, Ankit-Kumar Jitani, Jayanta Medhi

**Affiliations:** 1 *Department of Pathology, Post Graduate Institute of Medical Education and Research, Chandigarh, India.*; 2 *Department of Pathology, North Eastern Indira Gandhi Regional Institute of Health and Medical Sciences, Shillong, India.*; 3 *Department of Otorhinolaryngology, North Eastern Indira Gandhi Regional Institute of Health and Medical Sciences, Shillong, India.*

**Keywords:** EBV, EBER, ISH, Nasopharynx, Nasopharyngeal cancer

## Abstract

**Introduction:**

Nasopharyngeal carcinoma (NPC) is a common cancer in the North-East region of India. Though the role of environmental contributors of NPC in the North-Eastern part of India is firmly established, EBV as an etiological agent in the region remains unexplored.

**Material and Methods:**

Fifty-one patients, who presented at the department of ENT, NEIGRIHMS and were confirmed as NPC upon histopathological examination, were included in the study. Chromogenic in-situ hybridization (CISH) was used for the evaluation of EBER (Epstein Barr Virus Encoded RNA). Presence of nuclear signals was taken as positive for EBER expression. EBER status was correlated with various clinicopathological parameters like age, sex, dietary habits, histological types of NPC, and ethnicity of the patients.

**Results:**

The age range of the study group was 25 to 70 years with a mean age of 44.64 years and a male:female ratio of 3:2. Non-keratinizing undifferentiated type of NPC was the most common histological type. EBV was positive in 59% (30/51) of our cases. It showed a statistically significant correlation with the Naga community (P=0.01), with consumption of smoked food (P=0.02), and cigarette smoking (P=0.02). There was no correlation of EBV with age, sex, lymph node metastasis, stage, and histology.

**Conclusion:**

Our result indicates that EBV may be an additional risk factor in the pathogenesis of NPC in this region of India. So apart from lifestyle modification, a future study for a screening test for EBV viral load even in asymptomatic patients may be considered, for determination of disease susceptibility, early diagnosis, and proper management.

## Introduction

Nasopharyngeal carcinoma (NPC) is the most common cancer originating in the nasopharynx. It differs significantly from other cancers of the head and neck in its occurrence, causes, clinical behavior, and treatment (1). The global incidence of nasopharyngeal cancer in developed and developing countries are 0.6% and 2.1% respectively (2). In India, the incidence of NPC is 0.4%, with males being involved three times more commonly than females (3). Compared to other parts of India, the North-Eastern region has higher incidence of NPC (5.1 per 100000) (1). The North Eastern region of India represents a complex mixture of various ethnic groups and, among all the tribes, the mongoloid race has shown an increase in NPC incidence. The morbidity and mortality associated with this disease is a cause of major concern in this region (4).

NPC has a complex interaction of genetic; viral, like Epstein Barr Virus (EBV); environmental; and dietary factors which may be associated with the etiology of this disease (4). 

A modified WHO classification was proposed in 1991, which stratified NPC tumours into two histological groups: keratinizing squamous-cell carcinomas and non-keratinizing carcinomas (5). The latter was then subdivided into differentiated and undifferentiated carcinomas. The criterion used in this classification significantly correlated with EBV and non-keratinizing carcinoma had a stronger association with EBV infection (6). 

From the prognostic point of view, the undifferentiated type has a better prognosis than the keratinizing type of NPC, which means that the tumour associated with EBV has a better prognosis (7)**.** However, there are no studies undertaken in this region to know the exact prevalence of EBV in NPC and their relation with other clinicopathological parameters. Also, the social and dietary habit in this region is distinctly different from the rest of India, which could be a factor relating to the high incidence of NPC in this region. With this background, this study was undertaken with the aim to analyze Epstein Barr virus encoded RNA (EBER) expression in NPC and to evaluate the association of EBER status with various socio-clinico-pathological parameters of patients.

## Materials and Methods

This study was conducted in NEIGRIHMS, Meghalaya India. Being a tertiary care referral centre, our institute caters to the entire north eastern part of India, so the many ethnic communities in this region were almost equally represented. An approval from the institutional ethical committee for research involving human subjects was obtained before the study was conducted.

The study was undertaken from January 2013 to July 2014. All newly diagnosed NPC patients (n=51) with histopathological confirmation on punch biopsy sample and who received treatment in our institute during the study period were included as study group. Patients who were diagnosed before the period of the study but presented for radiotherapy treatment or for follow up during the study period were excluded. Patients with a history of concurrent or previous cancer in other organs and patients who had received any form of cancer therapy in the past were excluded. Patient history was collected prior to the beginning of treatment in all the cases and this included the age, gender, clinical, dietary, and social habits. Patients were stratified into user and non user depending on the dietary habit. Non users included the cases of NPC who did not give a history of intake of smoked food, or a history of cigarette smoking or alcohol abuse from last one year.

Among all the patients who satisfied the inclusion criteria, a punch biopsy specimen from the nasopharynx was collected in the department of Otorhinolaryngology and sent to the department of Pathology, NEIGRIHMS, for histopathological confirmation. All the tissues were fixed in 10% neutral buffered formalin, followed by tissue processing and sectioning of paraffin embedded tissue blocks. Morphological evaluations of cases were established by histopathological examination of Hematoxylin and Eosin stained paraffin block sections.

*Detection of EBER by chromogenic in-situ hybridization*

Formalin fixed and paraffin-embedded (FFPE) sections were evaluated for EBER using CISH (Zytovision, Germany). Sections of 5 μm thickness were taken on poly L-lysine coated slides, dewaxed in xylene, rehydrated in graded alcohol, incubated in 3% H_2_O_2_, and washed. Pepsin digestion was done for 10-20 minutes at 37˚C in a humidity chamber, followed by heat pre-treatment in EDTA at 95˚ C for 15 minutes. Ten μl of EBER probes were pipetted onto each sample and were covered with a cover slip and edges sealed with rubber cement. The samples were then denatured at 75˚C for 5 minutes on a hot plate and then transferred to a humidity chamber and hybridized at 37˚C for 60 minutes. Cover slips were removed, followed by serial application of Mouse-anti-DIG, anti-Mouse-HRP-polymer, and diaminobenzidine chromogen (DAB). EBER positivity was evaluated in the nucleus of the cell. Any brown coloured dot like signal from the nucleus of the cell was considered positive for EBV expression. Cases showing absence of this nuclear signal were considered negative for EBV (8). 

ZytoFast (+) DNA probe provided by the manufacturer were applied to the NPC blocks to check the nucleic acid integrity within the nucleus of the cells. Strong hybridization signal within the nucleus (for the DNA probe) of the cells verified the integrity of cellular nucleic acid material within the specimen. Also, 10 patients with histopathological diagnosis of nasal polyp were stained for EBER, which acted as a technical negative control for EBV and validated the technical aspect of the staining. With every batch of NPC cases, 1 negative control sample was run.

*Statistical analysis*

EBER status in nasopharyngeal carcinoma was evaluated by chi-square test. EBV status was compared with the demographic, social, and dietary habits of the patients and the histopathological type of the tumour. The test was considered to be statistically significant when their p values were < 0.05.

## Results

**Fig 1:**
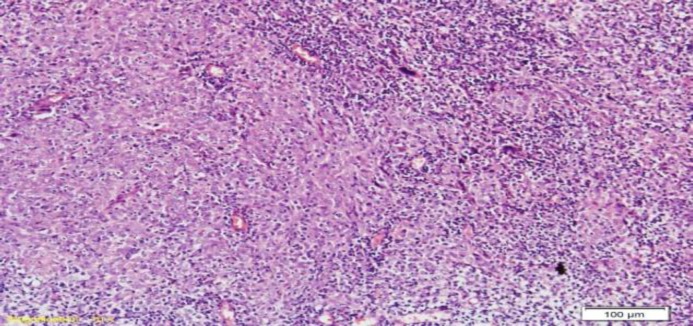
A case of non-keratinizing undifferentiated NPC, showing poorly differentiated tumour cells intricately mixed with lymphoid cells. (Haematoxylin & Eosin stain; 100x)

**Fig 2:**
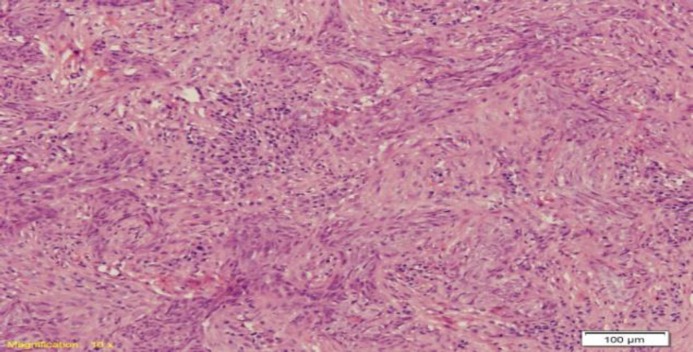
A case of non-keratinizing differentiated NPC showing nests and sheets of malignant squamous cells. These cells lack intracytoplasmic keratinization. (Hematoxylin & Eosin stain 100x

Of the 51 cases of NPC, there were 36 (71%) males and 15 (29%) females with a ratio of 2.4:1. The age of the patient varied from 25 to 70 years with a mean age of 44.64 years. Histologically, 45 (88%) cases were diagnosed as non-keratinizing undifferentiated types and 6 (12%) cases were diagnosed as non-keratinizing differentiated types (Figs 1,2). None of the cases were reported as keratinizing differentiated type.

**Table 1:** Patient characteristic and EBER association with various clinicopathological parameters

**Patient or tumour Characteristic**		**Total (n=51)**	**EBER Positive**	**EBER Negative**	**P Value**
Sex	Male	36 (70.58%)	22 (43.14%)	14 (27.45%)	0.6071
	Female	15 (29.42%)	08 (15.69%)	07 (13.73%)	
Age	21-40 yr	19 (37.26%)	11 (21.57%)	08 (15.69%)	0.4146
	41-60 yr	26 (50.98%)	14 (27.45%)	12 (23.53%)	
	61-70 yr	06 (11.77%)	05 (9.81%)	01 (1.96%)	
Smoked food	User	30 (58.83%)	28 (54.91%)	02 (3.93%)	0.0234
	Non user	21 (41.18%)	14 (27.45%)	07 (13.73%)	
Cigarette Smoking	User	30 (58.83%)	18 (35.30%)	12 (23.57%)	0.0269
	Non user	21 (41.18%)	06 (11.77%)	15 (29.42%)	
Alcohol	User	30 (58.83%)	16 (31.38%)	14 (27.45%)	0.1927
	Non user	21 (41.18%)	15 (29.42%)	06 (11.77%)	
Ethnicity	Naga	30 (58.83%)	24 (47.06%)	06 (11.77%)	0.0158
	Non-Naga	21 (41.18%)	10 (19.61%)	11 (21.57%)	
Cervical Lymph Node	Metastasis present	37 (72.55%)	22 (43.14%)	15 (29.42%)	0.8808
	Metastasis absent	14 (27.45%)	08 (15.69%)	06 (11.77%)	
Histological types	Non-keratinizing undifferentiated	45 (88.24%)	25 (49.02%)	20 (39.22%)	0.3806
	Non-keratinizing differentiated	06 (11.77%)	05 (9.81%)	01 (1.96%)	
					

**Fig 3:**
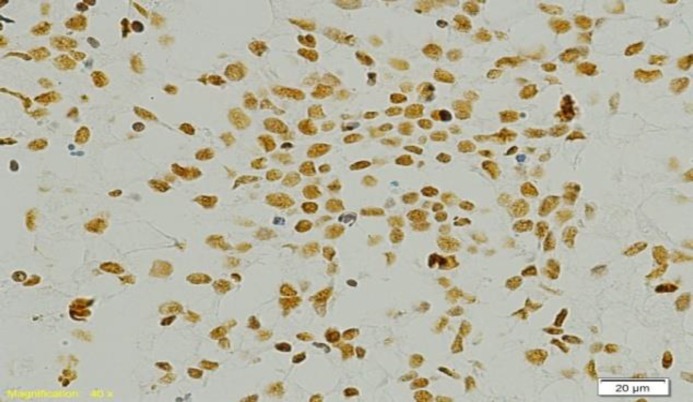
Positive Epstein Barr virus encoded RNA expression by chromogenic in situ hybridization. Brown dot like nuclear positivity is seen in tumour cells (in situ hybridization; 200x

**Fig 4:**
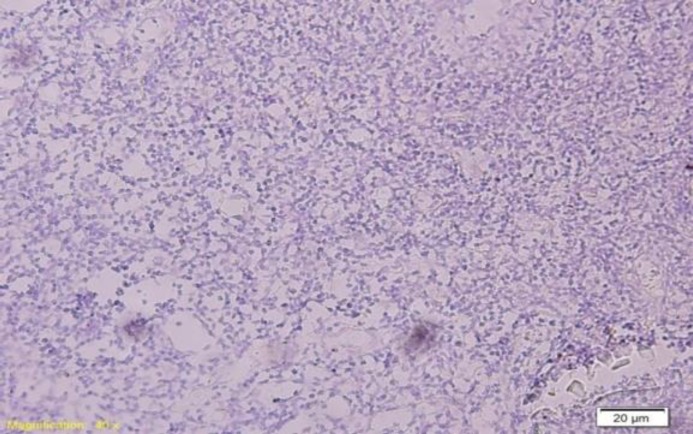
Negative expression of Epstein Barr virus encoded RNA by chromogenic in situ hybridization technique. No nuclear signal is seen (in situ hybridization; 100x

Table 1 shows the demographic and clinical details of patients and the tumour characterstics. Out of 51 cases, 30 (59%) cases were positive for EBER (95% confidence interval: 53.14% - 64.86%) and 21(41%) cases were negative (Figs 3,4).

The relationship between EBER and various clinicopathological parameters were statistically analysed. There was significant association of EBER expression with smoked food (P=0.02), Naga ethnic community (P= 0.01), and cigarette smoking (P= 0.02). Fifty five percent (n=28/51) of patients ingesting smoked food showed EBER positivity compared to 27% (n=14/51) of patients who did not ingest smoked food on a regular basis. In terms of ethnicity, we observed that EBER positivity was found in 47% (n= 24/51) cases of Naga patients and 20% (n=10/51) cases of non Naga patients. Thirty five percent (n=18/51) of smokers showed EBER positivity compared to 12% (n=6/51) of non smokers. There was no significant association of EBER with age, sex, lymph node metastasis, histology, and alcohol consumption. 

## Discussion

NPC is the most common cancer originating in the nasopharynx. It arises from the epithelial lining of the nasopharynx and comprises the vast majority of nasopharyngeal cancers in both high and low incidence populations (9).

In most regions of the world, NPC is a rare malignancy with an incidence of less than 1 per 100,000 people per year. NPC is uncommon even in the Indian subcontinent except in the north-eastern region of the country. The latest National Cancer Registry Programme reported the highest age-adjusted incidence rates of NPC in North Eastern states compared to other parts of this country (1).

In almost all surveyed populations, the incidence of NPC is two to three-folds higher in males compared to females. In high-risk groups, the incidence peaks at around 50 to 59 years of age and declines thereafter. Ellen T. et al. reported a minor incidence peak among adolescents and young adults (9). 

In most areas where NPC is endemic, EBV infection is presumed to occur at an early age (10). EBV is a ubiquitous human gamma herpes virus that was recognized and designated in 1964. EBV infects more than 90% of the adult population of the world. Following primary infection, the individual remains a lifelong carrier. Saliva is the main vehicle for EBV transmission from individual to individual, whereas organ and bone marrow transplantation is another risk for EBV infection. In NPC, EBV shows type ii latency where one can detect latent membrane protein antigen (LMP), EBER, and EBNA molecule within an infected cell (11). 

 In developing countries, EBV infection typically occurs during early childhood and is often asymptomatic, whereas in developed countries, infection often occurs during adolescence or adulthood. In our study, 27% of patients with an age range of around 41-60 years and 10% with age range of around 61-70 years were positive for EBV. We did not get any significant association of EBV with the age of the patients.

EBER was found to be positive in 59% of our cases. Out of all the positive cases, 83% of cases were non keratinizing undifferentiated type and 17% of cases were non-keratinizing differentiated type. None of the cases were of the keratinizing type. Though a previous study done by Smriti M. et al. and Pathmanathan R. et al. suggested an association between EBV and some histological subtypes of NPC, we did not find any significant association between them (6,12).

Sharma et al. in their study done in the North Eastern part of India reported that smoked meat and smoked fish are widely consumed as a major component of meals by the patients suffering from NPC (1). In the present study, the dietary pattern in the majority of patients (89%) was unhealthy. It comprised of smoked food and a minimal amount of fruits and vegetables. Smoked food was consumed by a majority of the patients (82%) suffering from NPC on a regular basis, in both males and females. In the pathogenesis of NPC, smoked food acts as a major contributing factor because of the release of nitrosamines, which act as a potent carcinogen. We found a statistically significant association between EBV infection and smoked food consumption in patients with NPC, establishing EBV as an additional risk factor for NPC in this part of India. 

Forty-seven percent of patients smoked cigarettes and 60% consumed alcohol in our study. We observed a significant association between EBV and smoking but not with alcohol consumption. These results are in accordance with the study performed by Cheng YJ et al. (13). Ethanol has been thought to be the key compound responsible for the effect of alcoholic beverage consumption on cancer. Chronic alcohol consumption also induces cytochrome P450 enzyme (CYP2E1) activity in mucosal cells. Induction of CYP2E1 can lead to stimulation of free radical formation and thus cause cell injury (14). Thus, smoking and EBV might have a synergistic effect in the pathogenesis of NPC.

In the study done by Kataki AC et al., it was seen that in the North-Eastern part of India, NPC was more common in mongoloid races (15). In the present study, out of 51 cases of NPC, 34 (67%) cases were from the Naga community, one of the mongoloid tribes of India intrinsic to the state of Nagaland. EBER has a significant association with this tribe. High incidence of NPC in the Naga community could be due to the overindulgence of this tribe in smoked food. The genetic makeup of the tribe along with EBV status might also play a significant role in the pathogenesis of NPC. Little is known about the genetic constituent of this tribe. Future research may be considered to know the exact genotype and its association with EBV in this region. Consumption of smoked food, smoking cigarettes, and drinking alcohol are significant contributing factors, modifying the multistage process of carcinogenesis of NPC, which might be amplified by the presence of EBV.

Analysis of EBV can be done by various methodologies, which includes in-situ hybridization, polymerase chain reaction, and immunohistochemistry for LMP. PCR can be applied for viral detection in formalin-fixed tissue, but the high sensitivity of PCR and the inability to localize the particular infected cells (epithelial or non-epithelial) renders the technique somewhat problematic in its application to tumours arising from mucosal surfaces from which EBV is regularly shed, even in normal people without malignancy. In situ hybridization has the virtue of identifying the infected cells and it can localize the virus within the cell. EBER1 transcript, despite its small size, is an appropriate target for in situ hybridization of clinical specimens where EBV associated epithelial cell malignancy is suspected. Because EBER transcripts are naturally amplified, they represent a reliable target for detecting and localizing EBV in tissue sections by in situ hybridization (16,17). Possibilities might arise that hybridization techniques might give false negative results in paraffin embedded tissue sections, but a study done by Hanel P. et al. found that the inability to detect EBERs in a proportion of tumour cells in a paraffin embedded tissue is not due to a down-regulation of gene expression but to a real absence of EBV from these cells. Those cases, which show negative results with the in situ hybridization method, also demonstrate an absence of EBV DNA with nested PCR technique. Thus, in situ hybridization could be a reliable tool in detecting EBV antigen in paraffin embedded tissue (18). 

Brousset P. et al. compared the in situ hybridization technique with an immunohistochemistry method for detecting EBV antigen in nasopharyngeal carcinoma (19). They came to a conclusion that in situ hybridization with EBER oligonucleotides, appears to be more reliable than immunohistochemistry with anti-latent membrane protein antibody to detect EBV in NPC in routine pathology (19). Tzyy-Choou Wu et al. also found that EBER1 transcript is an appropriate target for in situ hybridization detection of EBV in formalin-fixed paraffin- embedded carcinoma specimens (20). 

Antti A. et al., found 81% EBER positivity in NPC with CISH technique (21). They concluded that EBER positivity is associated with improved survival for NPC patients and p16 expression is reduced or absent in the majority of patients with NPC, and this appears to be a predictor for worse survival. Rajadurai Pathmanathan et al., found 100% positivity in NPC while using in situ hybridization (12). In comparison to other studies, our study showed EBER positivity in 59% cases. On the basis of this finding, we can suggest that socio-dietary factor, as previously discussed, forms a major risk factor for the development of NPC in this region. EBV may act independently or synergistically to further increase the risk associated with NPC development. 

## Conclusion

Studies so far suggest that development of NPC might be due to a complex interaction between genetic factors, exposure to carcinogens, and viral infections. The present study reveals the association of EBV with various clinicopathological parameters. Our result indicates that EBV is an additional risk factor for NPC in this region. So, apart from lifestyle modification, a future study for a screening test for detecting EBV viral load, in asymptomatic individuals of the high risk ethinicity, and a genetic study of these populations may be considered since knowing the disease susceptibility and making an early diagnosis, might have a profound effect on disease management and prognosis.
